# Moments of Change in Group Psychotherapy: Exploring Therapeutic Factors and Relational Processes

**DOI:** 10.3390/bs16081418

**Published:** 2026-08-18

**Authors:** Konstantinos Chelios, Ilias I. Vlachos

**Affiliations:** Department of Psychology, The American College of Greece, 12135 Athens, Greece; ivlachos@acg.edu

**Keywords:** emotional holding, group cohesion, group psychotherapy, moments of change, psychological insight, reflexive thematic analysis, relational processes, therapeutic factors, universality

## Abstract

Transformative experiences are often described as pivotal moments of change in psychotherapy, yet relatively little is known about how such experiences are perceived within group settings. This study explored how group members and therapists describe moments of change in group psychotherapy and examined the therapeutic factors and relational processes associated with these experiences. Eight semi-structured interviews were conducted with four former group members and four licensed group therapists. Data were analyzed using reflexive thematic analysis, while descriptive counts were used to illustrate the prominence of Yalom’s therapeutic factors in participants’ accounts. All eleven of Yalom’s therapeutic factors were identified in participant narratives, with universality and cohesion emerging most frequently. Moments of change were described as emotionally significant relational experiences characterized by shared humanity, recognition, and meaning-making. Five relational processes were identified: emotional holding, risk-taking and courage, internalization of the group, therapist emotional presence, and liberation from shame. Findings suggest that moments of change emerge through the interplay of relational safety, attuned therapist presence, and courageous interpersonal engagement. The study highlights relational processes through which established therapeutic factors become experientially meaningful and contribute to psychological change within group psychotherapy and may be associated with psychological insight.

## 1. Introduction

In every therapeutic encounter, most moments pass unnoticed—and then there are those that change everything. A glance, a shared silence or a timely sentence can initiate profound transformation (Hilzinger et al., 2021). Group psychotherapy is especially rich in such experiences. Within its dynamic relational field change often emerges not through sudden breakthrough but through subtle shifts in how members understand themselves and others (Lewin, 1951; Haas, 2024).

Since its early roots in medical settings (Pinney, 1978), group psychotherapy has been established as an effective treatment across diagnostic presentations (Barkowski et al., 2020; Burlingame et al., 2016). Members enter a “social microcosm” in which interpersonal patterns unfold in the here-and-now (Ends & Page, 1959). Within this relational atmosphere, participants often encounter turning points—emotionally charged interactions that deepen meaning, soften defenses and open new relational possibilities (Yalom & Leszcz, 2020).

Although group therapy research strongly supports its effectiveness, less is known about the lived experience of moments of change as perceived by group members and therapists. Concepts such as Stern et al.’s (1998) “moments of meeting” and insight-based “Aha” experiences (Kounios & Beeman, 2014) highlight relational catalysts, yet empirical studies rarely ask members or therapists to directly identify and describe such experiences (Shen et al., 2015).

Yalom’s eleven therapeutic factors—cohesion, universality, instillation of hope, altruism, interpersonal learning, imitative behavior, catharsis, existential factors, corrective recapitulation of the primary family group, imparting information and development of socializing techniques—remain foundational for understanding mechanisms of change in groups (Behenck et al., 2017; Ponomarenko et al., 2023; Sousa et al., 2020). While cohesion is often viewed as central (Marmarosh et al., 2005; Burlingame et al., 2018), research shows that the salience of different factors varies depending on group composition and context (Brandão et al., 2019; Wills et al., 2018). This variability underscores that change is dynamic and context-dependent and suggests the value of investigating how factors—and potentially additional processes—manifest in lived therapeutic experience.

Insight has been conceptualized in different ways across psychiatric and psychotherapeutic traditions. In severe psychopathology, particularly psychotic disorders, clinical insight refers to awareness of having a mental disorder, recognition of symptoms, understanding their consequences, and acknowledgment of the need for treatment (David, 1990; Vlachos et al., 2023; Vlachos, 2026). In contrast, psychotherapy has traditionally emphasized psychological insight, namely the capacity to achieve meaningful understanding of one’s emotions, relational patterns, internal conflicts, and personal narratives (Jennissen et al., 2018). Although both forms of insight involve increased self-awareness, they differ in focus: clinical insight concerns awareness of illness, whereas psychological insight concerns meaning-making and transformation of subjective experience. Group psychotherapy may constitute a particularly fertile context for the emergence of psychological insight, as individuals come to understand themselves through emotionally significant encounters with others. Moments of change may therefore represent relational contexts in which psychological insight is not simply acquired cognitively, but co-constructed within the interpersonal field of the group.

Importantly, psychological insight should be distinguished from Yalom and Leszcz’s (2020) therapeutic factor of self-understanding. While self-understanding refers to increased awareness of one’s interpersonal patterns, motives, or emotional responses, psychological insight constitutes a broader and more integrative process. It encompasses understanding the associations between past and present experiences, recurring relational patterns, emotional experiences, and psychological difficulties (Jennissen et al., 2018). Beyond cognitive recognition, psychological insight involves emotional meaning-making and transformation in the way individuals experience themselves in relation to others. In this sense, self-understanding may be viewed as one pathway through which psychological insight develops, rather than an equivalent construct.

At a broader level, moments of change are central to Change Process Research (CPR) (Elliott, 2010), which seeks to understand mechanisms of psychotherapeutic change (Burlingame et al., 2016). Drawing on participants’ own accounts positions them as “expert witnesses” of their experience (Elliott, 1984), helping bridge research and clinical practice (Duarte et al., 2019; Gullo et al., 2022). In the present study, moments of change refer to subjectively meaningful in-session experiences retrospectively identified by participants as catalysts of psychological change and transformation, rather than formally coded significant events derived from session-by-session process analysis.

While Yalom and Leszcz’s (2020) therapeutic factors describe broad mechanisms associated with group effectiveness, the experiential enactment of these mechanisms within specific in-session moments remains underexplored. The present study does not seek to introduce additional therapeutic factors. Rather, it explores the phenomenological micro-processes through which established mechanisms become subjectively meaningful and contribute to moments of change within group psychotherapy. In doing so, it contributes to CPR by articulating experiential micro-processes through which broader therapeutic mechanisms become subjectively enacted.

The present study addresses the following research questions:

(1) How do group members and therapists describe in-session moments of change in group psychotherapy?
(2) Which therapeutic factors, relational processes and broader patterns of meaning are associated with these moments of change, as reflected in participants’ lived experience?

## 2. Materials and Methods

### 2.1. Research Design

A qualitative design using reflexive thematic analysis (Braun & Clarke, 2006, 2021) was employed for its flexibility in capturing emotional and relational processes (Meier et al., 2008). Situated within an experiential qualitative framework, the analysis focused on how participants made meaning of moments of change within group psychotherapy rather than testing predefined constructs (Willig, 2013). In line with reflexive thematic analysis (Braun & Clarke, 2021), knowledge was understood as co-constructed through interaction between participant narratives and researcher interpretation. Rather than attempting to bracket assumptions, the researcher engaged in ongoing reflexive examination of interpretive positions and analytic decisions throughout the process.

### 2.2. Participants and Eligibility Criteria

Four former group members and four licensed group therapists participated (demographics in Table 1). Members had completed at least one year in ongoing psychotherapy groups but were no longer in treatment, facilitating reflection. Therapists had over three years of experience facilitating groups. This sample was purposive and information-rich. Groups varied in setting (private practice, psychiatric hospital), size (4–8 members) and orientation (group-analytic, psychodynamic, person-centered, Non-Directive Intervention). Interviews with therapists provided context, but analytic focus remained on members’ lived experience. The interview guide is presented in Appendix A.

### 2.3. Recruitment and Procedure

Participants were recruited through outreach to group-therapy networks, which disseminated the participant information sheet. Eight individuals expressed interest and met the inclusion criteria; all were interviewed. No individuals who expressed interest declined participation, and no participants were excluded. Written informed consent was obtained prior to interviews.

### 2.4. Data Collection

Semi-structured interviews invited rich descriptions of transformative group moments. Example prompts included “Can you describe a moment in group therapy that felt transformative?”, “What was happening in the group then?”, and “How did it affect you afterward?”. All of the interview questions are provided in Appendix A. Although interview questions invited participants to describe experiences they perceived as transformative, these experiences are conceptualized in the present study as moments of change within the broader framework of Change Process Research.

Although former group members constituted the primary source of lived experiential data, therapists were included as reflective informants capable of describing and contextualizing moments of change observed across their clinical work. Including both perspectives allowed the phenomenon to be explored from complementary viewpoints while acknowledging their distinct epistemic positions. Former group members provided first-person accounts of lived experience, whereas therapists contributed reflective clinical perspectives that contextualized and enriched understanding of relational processes. Similar multi-perspective qualitative designs have been used in psychotherapy process research to deepen understanding of therapeutic change while recognizing that clients and therapists occupy different experiential positions (Donald et al., 2018). More recent work examining significant therapeutic events has likewise demonstrated that patients’ and therapists’ perspectives are both convergent and complementary, each contributing unique insights into therapeutic processes (Leduc et al., 2026). The analytic focus therefore remained on members’ lived experience, while therapist narratives served primarily to contextualize, illuminate, and triangulate the experiential phenomena identified across the dataset rather than generate a separate thematic structure. All interviews were conducted in Greek. Quotations presented in this manuscript were translated into English by the first author. Particular attention was paid to preserving participants’ intended meaning, emotional tone, and contextual nuance, while ensuring clarity and readability in English. The therapist interview guide included a question inviting therapists to reflect on the role of Yalom and Leszcz’s (2020) therapeutic factors in moments of change. Because therapists participated as experienced clinicians capable of reflecting on their observations through an established theoretical framework, this question was intended to facilitate deeper professional reflection rather than structure the analytic process. This approach is consistent with qualitative interviewing, which recognizes interviews as knowledge-producing interactions in which theoretically informed questions may facilitate reflective dialogue with expert participants (Kvale & Brinkmann, 2009). Former group members were not asked about Yalom’s therapeutic factors. Interviews lasted 20–35 min, were audio-recorded, anonymized, and transcribed verbatim.

### 2.5. Analysis

Reflexive thematic analysis followed Braun and Clarke’s (2006, 2021) six phases. The dataset generated approximately 300 coded extracts, which informed the development and refinement of the themes. Initial codes were generated inductively through close and repeated engagement with the interview transcripts. Segments of text describing participants’ experiences of moments of change were coded in a flexible and iterative manner, primarily at a semantic level while remaining open to latent relational meanings where these were implied. Coding was recursive; codes were refined, collapsed, or expanded as patterns of shared meaning across participants became visible through ongoing comparison with the dataset. Through this process, related codes were gradually clustered into candidate themes that captured broader patterns in participants’ accounts. These candidate themes were subsequently reviewed against the full dataset and refined to ensure conceptual coherence, internal consistency, and clear distinctions between themes, with extracts revisited iteratively to refine thematic boundaries and confirm that themes captured patterned meaning across participants rather than isolated instances.

Consistent with reflexive thematic analysis, theoretical interpretations extending beyond explicit semantic content were developed iteratively throughout the analytic process. Psychological insight was not treated as a predefined analytic category or explicitly explored during data collection; rather, it emerged as a theoretically informed interpretive concept during the integration of themes, relational processes, and participants’ accounts.

Thematic development was primarily inductive and data-driven. Yalom’s therapeutic factors were not employed as an a priori coding framework. Rather, once themes had been developed through engagement with participants’ narratives, relevant extracts were subsequently mapped onto Yalom’s conceptual model to explore areas of convergence and divergence between participants’ accounts and established group psychotherapy frameworks. This mapping served as a theoretical dialogue with existing frameworks rather than as a deductive analytic structure. Table 2 presents illustrative examples of how analytically coded meaning units were subsequently mapped onto Yalom’s therapeutic factors to generate the descriptive frequencies reported in Table 3, thereby enhancing analytic transparency.

Although not intended for statistical generalization, simple counts were recorded for Yalom’s factors to enhance analytic transparency regarding their representation across the dataset. The reported frequencies represent analytic coding instances (i.e., coded meaning units) rather than the number of participants. Each meaningful segment reflecting a therapeutic factor or relational process was coded independently; consequently, multiple excerpts from the same participant contributed to the descriptive count when they represented distinct analytic instances. These frequencies are presented solely as heuristic indicators of descriptive salience and are not intended to indicate prevalence, statistical significance, or the relative importance of themes. Consistent with reflexive thematic analysis, theme development and interpretation were guided primarily by the richness, coherence, and interpretive value of the data rather than numerical frequency (Braun & Clarke, 2021; Byrne, 2022).

### 2.6. Reflexivity and Analytic Rigor

Reflexivity was an integral part of the research process. The interviews were conducted by the first author, a psychologist with four years of training in group formats and ongoing advanced training in group psychotherapy. This professional background and theoretical positioning inevitably informed the analytic process by shaping sensitivity to relational dynamics, attunement, and group processes. Consistent with reflexive thematic analysis, these perspectives were not treated as sources of bias to be eliminated but as interpretive resources that were engaged reflexively throughout the analysis (Braun & Clarke, 2022). Reflexive notes documented assumptions, emotional reactions, and evolving interpretations throughout the analytic process (Etherington, 2017). Regular supervision supported critical reflection, methodological integrity, and transparency while ensuring that interpretations remained closely grounded in participants’ accounts (Levitt et al., 2018; Smith et al., 2021).

### 2.7. Ethical Considerations

Ethical approval for this study was obtained from the Institutional Review Board of the American College of Greece. All participants provided written informed consent prior to participation. Participation was voluntary, confidentiality was maintained throughout the research process, and identifying information was removed during transcription and reporting.

Given the retrospective and potentially emotionally evocative nature of the interviews, particular attention was paid to participants’ emotional safety. Former group members were informed that participation was entirely voluntary and that they could pause or discontinue the interview at any time without explanation or consequence. Participants were also reminded that they were free to decline answering any question that felt distressing or overwhelming. No participants requested termination of the interview, and no significant distress was observed during data collection.

## 3. Results

### 3.1. Presence and Descriptive Frequency of Yalom’s Therapeutic Factors

In line with the qualitative and non-generalizable nature of reflexive thematic analysis, counts are presented to illustrate descriptive prominence and analytic transparency rather than statistical generalization or relative importance. Frequencies for all eleven therapeutic factors appear in Table 3.

All eleven therapeutic factors described by Yalom and Leszcz (2020) were identified in participant narratives, albeit with varying descriptive prominence (see Table 3). Universality and group cohesion were most frequently described, followed by catharsis and existential factors. Imparting information and interpersonal learning appeared with moderate frequency, whereas instillation of hope, altruism, self-understanding, imitative behavior, and corrective recapitulation were less frequently referenced. Overall, moments of change were most often grounded in shared humanity, emotional connection, and meaning.

**Table 3:** Descriptive frequencies of Yalom’s therapeutic factors across participant interviews.

Therapeutic Factor	Frequency (n)
Universality	11
Group cohesion	9
Catharsis	7
Existential Factors	7
Imparting information	6
Interpersonal learning	6
Instillation of hope	5
Altruism	4
Self-understanding	4
Imitative behavior	2
Corrective recapitulation	2

*Note.* Counts are provided only as heuristic indicators of descriptive prominence within the dataset and are not intended to imply quantitative comparison or statistical significance.

### 3.2. Relational Processes Associated with Moments of Change

Five relational processes associated with moments of change were identified across participant accounts, capturing how moments of change were experienced in relational terms (see Table 4). Emotional holding was descriptively represented more frequently within the dataset, followed by risk-taking and courage and internalization of the group. Therapist emotional presence and liberation from shame appeared less frequently. Additional representative participant quotations illustrating each theme are provided in Appendix B.

#### 3.2.1. Emotional Holding: “A Place That Could Hold Me”

Emotional holding referred to participants’ experience of feeling emotionally contained, understood, and supported within the group, particularly during moments of vulnerability. Participants described moments of change as occurring when difficult emotions could be shared and carried collectively rather than endured alone.

While waiting for the court’s decision regarding the custody of her child, one participant described how the group became the only place where she felt emotionally supported throughout months of uncertainty and fear. Reflecting on one particularly difficult session, she recalled:

“It was like the group carried the weight with me … I didn’t feel alone with it for the first time.”

(P1)

Other participants described similar experiences of emotional holding in different therapeutic contexts:

“They didn’t rush me. They just stayed … That was the moment I realized I could trust them.”

(P2)

“Sometimes I felt the whole circle was holding something too heavy for one person to carry alone.”

(T1)

#### 3.2.2. Risk-Taking and Courage: “Choosing to Step Forward”

Risk-taking and courage described moments in which participants disclosed previously hidden experiences, expressed difficult emotions, or confronted fears despite uncertainty about how they would be received. Moments of change were frequently associated with these acts of interpersonal courage.

After struggling for several sessions with whether to disclose their transgender identity, one participant described the moment of finally speaking openly to the group as a decisive turning point in therapy:

“I can either stay silent or finally say it … and when I said it, everything changed.”

(P3)

Other participants and therapists similarly described moments of change emerging through different acts of interpersonal courage:

“My heart was racing … If I say this, everything might change.”

(P4)

“Every breakthrough started with someone daring to say what they thought they never would.”

(T1)

#### 3.2.3. Internalization of the Group: “Taking the Group Home”

Internalization of the group referred to participants’ experience of carrying the group’s supportive presence beyond the therapy room. Members described drawing upon imagined group responses and internalized relational experiences in their everyday lives.

While facing increasing pressure at work months after therapy had ended, one participant described instinctively asking themselves what the other group members would have said before deciding how to respond:

“Even outside the room, I would think: what would they tell me now?”

(P3)

Comparable experiences of internalizing the group were described across different life situations following the completion of therapy:

“My secret angel… the part that speaks kindly because they once did.”

(P1)

“Patients come back years later and say, ‘The group is still with me in my head.’”

(T4)

#### 3.2.4. Therapist Emotional Presence

Therapist emotional presence referred to participants’ experience of the therapist as emotionally attuned, authentic, and genuinely engaged. Moments of change were often linked to the therapist’s way of being rather than specific interventions.

After disclosing a severe traumatic experience from childhood, one participant described how seeing the therapist visibly moved by the disclosure became the first moment they truly felt that another person genuinely cared about their suffering:

“She looked at me with tears in her eyes… I knew she cared.”

(P4)

Other participants and therapists described emotionally attuned presence in different therapeutic moments:

“It wasn’t what he said. It was how he was with me.”

(P2)

“My presence mattered more than my perfect formulation.”

(T3)

#### 3.2.5. Liberation from Shame: “From Hiding to Being Seen”

Liberation from shame described experiences in which participants revealed aspects of themselves they expected would be rejected, only to encounter acceptance, recognition, and shared understanding. These moments often marked important shifts in self-perception and interpersonal trust. 

After revealing that they had been unfaithful in a previous relationship, one participant expected rejection and moral judgment from the group. Instead, the accepting responses of the other members fundamentally changed how they experienced shame and self-acceptance:

“I thought they’d reject me … but they nodded, and I felt human again.”

(P1)

Similar experiences of acceptance following the disclosure of different shame-related experiences were also described by other participants:

“I expected disgust or pity … What I met instead was recognition.”

(P4)

“I could almost feel the shame leaving the room when others said, ‘Me too.’”

(T1)

Although all five relational processes were identified across the dataset, participants varied in the aspects they emphasized as central to their moments of change. For some, transformation was primarily associated with emotional holding and acceptance, whereas others highlighted courageous self-disclosure, interpersonal feedback, or the therapist’s emotional presence. These variations suggest that moments of change may emerge through multiple relational pathways rather than following a single, uniform process. Additional participant quotations supporting each theme are presented in Appendix B.

## 4. Discussion

The findings reaffirm the relevance of Yalom and Leszcz’s (2020) therapeutic factors while showing that their prominence varies across group contexts. At the same time, the analysis identified five emergent processes that deepen our understanding of how transformation unfolds in lived relational experience. These findings suggest that Yalom and Leszcz’s (2020) therapeutic factors may operate at the level of macro-mechanisms of change, whereas the emergent processes identified here illuminate how moments of change are subjectively experienced and enacted within specific in-session interactions.

Although all eleven factors appeared in participants’ narratives, universality and group cohesion were most frequent. This differs from the traditional emphasis on cohesion as the central mechanism (Marmarosh et al., 2005; Burlingame et al., 2018). In this study, several participants entered therapy with heightened isolation and shame, which may explain why universality—realizing others feel like me—may have served as an important gateway to engagement. Similar patterns have been documented in groups facing stigma or identity-related challenges, such as cancer survivors, individuals recovering from alcohol use, and hearing-voices groups (Furnes et al., 2014; Greenfield et al., 2013; Ulman, 2002).

Cohesion remained closely linked to transformation, consistent with research connecting it to emotional safety and therapeutic engagement (Burlingame & Strauss, 2021). Catharsis and existential factors also played notable roles, particularly when embedded within supportive relational interactions, echoing previous findings that catharsis is most impactful within cohesive groups (Groos & Shakespeare-Finch, 2013; Johnson et al., 2006) and aligning with existential themes observed in psychotherapy (Vos et al., 2015).

Interpersonal learning and imparting information emerged in participants’ accounts of receiving feedback, recognizing relational patterns, and gaining new perspectives, paralleling research on insight and relational change in group therapy (Joyce et al., 2011; Brouzos et al., 2021). Instillation of hope, altruism, and self-understanding were less frequent but aligned with earlier literature on their supportive roles (Sayin et al., 2008). Imitative behavior and corrective recapitulation appeared rarely, consistent with contemporary findings.

Together, these results support the utility of Yalom’s framework while showing that the experiential core of moments of change was grounded in shared humanity, emotional connection, and meaning making. The five emergent processes offer an experiential perspective on the classical model by illustrating how transformation is subjectively experienced within the group, highlighting relational micro-processes not fully captured by Yalom’s broader therapeutic factors.

The present findings further suggest that moments of change may provide an important experiential context through which therapeutic change becomes subjectively meaningful. Rather than isolated events, they may reflect the convergence of multiple therapeutic factors within the relational field. Participants frequently described universality, cohesion, altruism, catharsis, interpersonal learning, and emotional holding as converging within single transformative experiences, giving rise to new meanings, emotional shifts, and altered relational expectations.

Building on this perspective, participants’ narratives also invited a broader interpretive understanding through the concept of psychological insight. Although not specified as a predefined analytic category or explicitly explored during data collection, psychological insight emerged through reflexive thematic analysis as a theoretically informed interpretation integrating self-understanding, relational meaning-making, and transformative change. Although self-understanding appeared relatively infrequently as a distinct therapeutic factor, participants often described not only recognizing aspects of themselves but also experiencing emotionally significant shifts in self-perception, relational meaning, and personal identity. Psychological insight therefore appeared to extend beyond Yalom and Leszcz’s (2020) conceptualization of self-understanding, reflecting the reorganization of emotional, interpersonal, and existential meanings within the relational matrix of the group. Consistent with Hazan et al. (2026), this insight appeared to develop gradually through repeated emotionally significant relational experiences.

### 4.1. Emergent Processes

Beyond Yalom’s factors, five relational processes captured additional dimensions of moments of change: emotional holding, risk-taking and courage, therapist emotional presence, internalization of the group, and liberation from shame. Although conceptually overlapping with established factors, participants described these processes as distinct in their lived experience. Rather than representing additional therapeutic factors, they may be understood as experiential micro-processes through which transformation was subjectively experienced within the relational context of the group.

Table 5 illustrates how the emergent relational processes relate to established therapeutic constructs. The intention is not to propose entirely novel therapeutic mechanisms but to offer a phenomenological refinement of existing concepts by describing how participants experienced these relational processes during moments of change in group psychotherapy. Accordingly, the proposed processes should be understood as complementary experiential formulations that enrich, rather than replace, existing theoretical models of therapeutic change.

An additional perspective for interpreting these findings concerns the increasing complexity of contemporary clinical populations. Contemporary conceptualizations suggest that patient complexity extends beyond diagnostic severity to encompass interacting psychological, interpersonal, social, and contextual factors that shape individuals’ therapeutic needs (Nicolaus et al., 2022). More broadly, recent discussions across healthcare argue that traditional explanatory frameworks may capture only part of this multidimensional complexity, highlighting the importance of relational and contextual dimensions of care (Cesare et al., 2026). Consistent with this perspective, contemporary psychotherapy has also emphasized the growing relational complexity of therapeutic work (Haslam, 2026). Within this context, the prominence these relational processes may reflect the increasing importance of relationally responsive therapeutic environments for individuals with multidimensional needs. Although the present study did not examine patient complexity directly, this perspective offers one possible theoretical framework for interpreting the present findings and warrants further investigation.

Taken together, these findings may also be understood through the concept of the therapeutic window (Cole & Barney, 1987). The identified relational processes appeared to create conditions in which emotionally significant experiences could be explored safely and meaningfully. From this perspective, moments of change may represent therapeutic windows within the relational field of the group.

The present findings further suggest that moments of change may represent privileged contexts for the emergence of psychological insight. Whereas clinical insight in severe psychopathology primarily refers to awareness of illness and need for treatment (Vlachos et al., 2023; Vlachos, 2026), the moments described by participants reflected a different form of insight: an emotionally grounded understanding of oneself, one’s relational patterns, and one’s place in relation to others. Importantly, such insight did not emerge as an exclusively cognitive achievement or solitary realization. Rather, it appeared to be co-constructed within the relational field of the group through experiences of universality, emotional holding, courageous disclosure, therapist attunement, and liberation from shame. From this perspective, moments of change may constitute the experiential synthesis through which multiple therapeutic factors converge and become psychologically meaningful.

#### 4.1.1. Emotional Holding

Emotional holding resonated strongly with classical and contemporary psychoanalytic perspectives (Winnicott, 1955; Bion, 1962; Slochower, 2011). Participants described the group as a relational container for overwhelming emotional states. This co-constructed holding created a depth of safety that extended beyond cohesion and warranted recognition as a distinct therapeutic process.

#### 4.1.2. Risk-Taking and Courage

Acts of courage—revealing shame-laden material, confronting fear, or speaking difficult truths—were repeatedly described as catalysts for change. Consistent with Shay’s (2021) conceptualization of courage as an emotionally risky decision without guaranteed outcome, these moments frequently preceded catharsis, connection, and enhanced self-understanding.

#### 4.1.3. Internalization of the Group

Internalization emerged as a particularly enduring process. Consistent with object-relations perspectives (Schermer, 2000; Rutan et al., 2014), participants described carrying the group’s voices, emotional stance, and relational imprint into everyday life, sometimes years after therapy had ended. This enduring psychological resource reflects transformation beyond the therapy room, as repeated group experiences become integrated into relational schemas shaping future perceptions of self and others (Baldwin, 1992).

From an attachment-informed perspective, this process may reflect the incorporation of corrective relational experiences into evolving internal working models (Mikulincer et al., 2003). These internalized representations may continue to shape expectations of recognition, safety, and mutuality across new interpersonal contexts, extending the group’s influence beyond therapy (Andersen & Chen, 2002).

Importantly, internalization extends beyond the immediate experience of cohesion by illustrating how shared group experiences become enduring psychological resources. Moments of change may therefore contribute not only to in-session change but also to a lasting reorganization of relational expectations.

#### 4.1.4. Therapist Emotional Presence

Participants emphasized the therapist’s authenticity, attunement, and shared affect as central to transformation. This aligns with studies showing that emotional responsiveness enhances alliance and emotional depth (Imel et al., 2014; Furrow et al., 2012; Abargil & Tishby, 2021). Therapists’ reflections echoed research on the importance of self-awareness and emotion regulation during high-affect moments (Putrino et al., 2020). Emotional presence thus emerged as an active mechanism of change.

#### 4.1.5. Liberation from Shame

Shame reduction functioned as another core mechanism. Echoing Shay (2021), participants described fear of humiliation as a major initial barrier; yet group processes—particularly universality and emotional holding—were uniquely capable of dismantling shame. Transformative moments often involved revealing long-hidden aspects of identity or trauma and receiving recognition instead of rejection. This relational disconfirmation of shame reflects shifts seldom achieved in dyadic therapy alone (Davidoff, 2002).

### 4.2. Influence of Therapeutic Approach

Despite the heterogeneity of therapeutic orientations represented in the sample (group-analytic, psychodynamic, person-centered, and NDI), participants described several comparable relational processes. This observation is consistent with Bhatt-Mackin and Leszcz’s (2022) proposition that core relational mechanisms, such as attunement, cohesion, and shared meaning, may operate across group psychotherapy models. However, given the exploratory design and heterogeneous sample, these findings should be interpreted cautiously rather than as evidence of common processes across all group psychotherapy models. Moments of change were consistently associated with relational rather than model-specific interventions, despite differences in therapeutic formats (e.g., creative techniques).

### 4.3. Methodological Considerations

The small, intentionally focused sample supports analytic depth while limiting transferability to structured or short-term groups. Consistent with qualitative research principles emphasizing information power over sample size (Malterud et al., 2016), the specificity of the research aim, the richness of the interview material, and the experiential focus justified a focused sample. Nevertheless, heterogeneity in therapeutic orientation, clinical setting, and professional role reduced sample specificity and limits the extent to which the identified relational processes may be interpreted as common across different models of group psychotherapy. Accordingly, the findings should be understood as exploratory and contextually situated rather than broadly transferable. In addition, participants volunteered to participate and may therefore represent individuals particularly willing to reflect on, or positively evaluate, their therapeutic experiences.

Several additional limitations should be acknowledged. Because participants reflected retrospectively on meaningful therapeutic experiences, their accounts may have been shaped by memory reconstruction and subsequent meaning-making, potentially privileging emotionally salient moments over more gradual or less memorable micro-processes of change. Consequently, cumulative or less emotionally salient therapeutic processes may have been underrepresented. Furthermore, the relatively brief duration of some interviews may have limited opportunities to explore divergent experiences or elaborate on less salient moments of change.

As in all qualitative interviewing, participants’ narratives may also have been influenced by social desirability and the co-constructed nature of the interview interaction. Moreover, because the study relied exclusively on retrospective interviews rather than direct observation of therapy sessions, it was not possible to examine moments of change as they unfolded in real time. Similarly, the absence of independent outcome measures prevented examination of whether reported moments of change were associated with measurable therapeutic improvement.

An additional consideration concerns the therapist interview guide. Although therapists were invited to reflect on Yalom and Leszcz’s (2020) therapeutic factors as part of a theoretically informed interview, this question may have increased the likelihood that established therapeutic factors were explicitly identified. Accordingly, the presence of all eleven therapeutic factors should be interpreted with methodological caution. Importantly, former group members were not asked about these factors, and the five emergent relational processes were not introduced through either interview guide but emerged inductively through reflexive thematic analysis across the complete dataset.

Finally, consistent with reflexive thematic analysis, the researcher’s clinical background and theoretical orientation inevitably informed the analytic process. Rather than being viewed as sources of bias to be eliminated, these were treated as interpretive resources and engaged reflexively throughout coding, theme development, and interpretation (Braun & Clarke, 2021, 2022). Reflexive practices were employed throughout the study to enhance transparency and methodological rigor.

### 4.4. Clinical Implications

The findings highlight the importance of fostering universality, emotional holding, and therapist attunement as relational foundations for moments of change. Rather than focusing solely on therapeutic techniques, therapists may benefit from attending closely to relational cues indicating that a participant is approaching a potentially transformative moment, such as increased emotional vulnerability, hesitation before disclosure, or emerging experiences of shame. In these moments, tolerating therapeutic silence, pacing interventions sensitively, and maintaining an emotionally attuned presence may facilitate courageous self-disclosure while preserving a sense of psychological safety. This interpretation is consistent with recent psychotherapy process research emphasizing the clinical importance of recognizing and remaining engaged with clients’ moment-by-moment experiences of change, as well as with contemporary perspectives highlighting therapist responsiveness and emotional attunement as central components of effective group facilitation (Solberg Kleiven et al., 2024; Davì et al., 2026).

Encouraging reflection on how the group experience may continue beyond therapy may further strengthen the internalization of therapeutic gains. Overall, attending to these relational micro-processes may enhance therapists’ ability to recognize and facilitate moments of change within the evolving group process.

### 4.5. Future Research Directions

Future work may examine moments of change across different stages of therapy or investigate cultural and identity-based influences on group processes. Micro-analytic studies using session recordings could capture subtle relational dynamics. The emergent processes—especially emotional holding, internalization and shame liberation—may warrant development of process-informed conceptual refinements or qualitative-derived measures.

Future studies could also investigate whether moments of change function as integrative therapeutic events that synthesize multiple therapeutic factors and whether their frequency or quality predicts therapeutic outcome. In addition, explicitly operationalizing psychological insight during study design and data collection would allow its relationship with moments of change to be examined more directly. Longitudinal and micro-process designs integrating qualitative methods with validated insight measures may further clarify how psychological insight develops through relational experience in group psychotherapy.

## 5. Conclusions

Moments of change in group psychotherapy emerged as emotionally significant relational experiences in which participants felt seen, held and accompanied. The five relational processes identified in this study—emotional holding, risk-taking and courage, therapist emotional presence, internalization of the group and liberation from shame—extend our understanding of how therapeutic change is experienced within groups. Rather than proposing additional therapeutic factors, this study refines our understanding of how established mechanisms become experientially meaningful in participants’ lived experiences. Findings further suggest that transformative moments may represent key relational contexts through which psychological insight emerges and therapeutic change becomes personally meaningful and enduring, potentially enabling participants not only to understand themselves differently, but also to experience themselves differently in relation to others. Even brief moments of connection, recognition, or vulnerability may generate relational shifts that endure beyond the therapy room, carried forward as internalized experiences that continue to shape participants’ relational lives.

## Figures and Tables

**Table 1:** Participants’ characteristics and group context.

Participant	Role	Age Range	Gender	Group Setting	Group Orientation	Duration in Group	Session Length
P1	Member	50–59	Female	Private Practice	Group-Analytic	>3 year	2 h
P2	Member	50–59	Male	Private Practice	Person-Centered	>1 year	1.5–2 h
P3	Member	30–39	Female	Private Practice	Psychodynamic	>2 years	1.5–2 h
P4	Member	60–69	Male	Private Practice	Non-Directive Intervention (NDI)	>2 years	2 h
T1	Therapist	50–59	Male	Private Practice	Group-Analytic	>5 years’ experience	2 h
T2	Therapist	50–59	Male	Private Practice	Group-Analytic	>8 years’ experience	2 h
T3	Therapist	50–59	Female	Private Practice	Person-Centered	>10 years’ experience	1.5–2 h
T4	Therapist	40–49	Female	Psychiatric Hospital	Group-Analytic	>2 years’ experience	2 h

*Note.* Members refer to former group therapy participants; therapists refer to licensed group psychotherapists facilitating groups at the time of the study.

**Table 2:** Illustrative analytic coding instances contributing to the descriptive frequencies of Yalom’s therapeutic factors.

Illustrative Participant Quotation	Meaning Unit/Initial Codes	Therapeutic Factor (Yalom & Leszcz, 2020)	Illustrative Coding Instance
“For the first time, I realized that everyone in the room carried struggles similar to mine. I no longer felt different.”	Shared experience; common humanity; reduced isolation	Universality	1 coding instance (n = 11)
“The group gradually became a place where I felt accepted exactly as I was, without needing to hide parts of myself.”	Sense of belonging; acceptance; emotional safety	Group cohesion	1 coding instance (n = 9)
“Listening to everyone else’s reactions helped me recognize patterns in myself that I had never noticed before.”	Self-reflection; increased self-awareness; new perspective	Self-understanding	1 coding instance (n = 7)
“The honest feedback I received changed the way I relate to people outside the group.”	Interpersonal feedback; relational learning; behavioral awareness	Interpersonal learning	1 coding instance (n = 7)
“Watching other members overcome similar difficulties made me believe that I could eventually change too.”	Hope through others; positive expectations; shared progress	Instillation of hope	1 coding instance (n = 5)
“When I finally expressed everything, I had kept inside for years, I felt an enormous emotional release.”	Emotional expression; release of affect; relief	Catharsis	1 coding in stance (n = 6)
“I left each session with practical ideas that I could immediately apply in my everyday life.”	Practical guidance; learning new strategies; acquiring knowledge	Imparting information	1 coding instance (n = 6)
“Supporting another member unexpectedly helped me understand my own experiences more deeply.”	Helping others; reciprocal growth; empathy	Altruism	1 coding instance (n = 4)
“Seeing another member speak openly encouraged me to take the same risk myself.”	Observational learning; modelling; encouragement	Imitative behavior	1 coding instance (n = 4)
“I suddenly realized I was responding to people exactly as I used to respond to my parents.”	Re-enactment of early relationships; family patterns; relational awareness	Corrective recapitulation of the primary family group	1 coding instance (n = 2)
“The group helped me accept that uncertainty, loss and vulnerability are avoidable parts of life.”	Acceptance of life realities; existential awareness; meaning-making	Existential factors	1 coding instance (n = 2)

*Note:* The quotations are verbatim participant excerpts illustrating individual analytic coding instances (coded meaning units). Descriptive frequencies reported in Table 3 represent coding instances rather than participants; therefore, multiple excerpts from the same participant could contribute separate coding instances. Frequencies are presented solely to enhance analytic transparency and should not be interpreted as participant counts, prevalence estimates, or indicators of thematic importance (Braun & Clarke, 2021; Byrne, 2022).

**Table 4:** Relational Processes Associated with Moments of Change.

Emotional holding	7
Risk-taking and courage	5
Internalization of the group	5
Therapist’s emotional presence	4
Liberation from shame	3

*Note.* Processes are presented in order of descriptive coding frequency within the dataset.

**Table 5:** Conceptual positioning of the emergent relational processes in relation to established therapeutic constructs.

Emergent Relational Process	Closest Existing Construct(s)	Phenomenological Refinement
Emotional holding	Containment (Bion, 1962), Group cohesion (Yalom & Leszcz, 2020)	While containment primarily refers to the therapist’s capacity to metabolize emotional experience and cohesion describes the sense of belonging within the group, emotional holding captures participants’ lived experience of feeling emotionally received, accepted, and psychologically held within the relational field during moments of change.
Risk-taking and courage	Self-disclosure; Interpersonal learning	Existing concepts describe disclosure and learning processes. This process emphasizes the subjective decision to become vulnerable despite anticipated fear, highlighting courage itself as an active psychological process preceding transformative change.
Therapist emotional presence	Therapeutic alliance; Empathy; Corrective emotional experience	Rather than reflecting the overall quality of the therapeutic relationship, this process refers specifically to participants’ experience of the therapist’s emotionally engaged, authentic, and attuned presence during transformative moments.
Internalization of the group	Group cohesion; Internalization; Corrective emotional experience	Whereas cohesion primarily operates within ongoing group participation, internalization of the group describes the enduring psychological continuation of the group after therapy has ended, functioning as an internal relational resource in participants’ everyday lives.
Liberation from shame	Universality; Acceptance; Corrective emotional experience	Universality reduces isolation through recognizing shared experience. Liberation from shame is differentiated by describing the experiential transformation of shame into self-acceptance through repeated experiences of relational acceptance, validation, and belonging.

*Note.* The table illustrates how the emergent relational processes relate to established therapeutic constructs while highlighting their phenomenological contribution to understanding participants’ lived experience of moments of change.

## Data Availability

Due to the qualitative nature of the study and the sensitive clinical material involved, the data are not publicly available in order to protect participant confidentiality.

## Appendix A. Interview Guide

For the participants (group members)

1. Can you share a specific moment during group therapy that felt transformative for you? What led to that moment? What happened during that moment?
2. How did you feel before, during, and after the transformative moment? What emotions did it evoke?
3. In what ways did the interactions with other group members contribute to your experience of transformation?
4. How has this transformative experience changed your perspective or behavior outside the therapy? Have you noticed any lasting effects?
5. Were there any challenges or barriers you encountered in the group that might have hindered transformation? How did you overcome them?

For the therapists

1. Can you recall a transformative moment you observed in the group?/What made that moment significant? What was your role in facilitating it?
2. In what ways do you see Yalom’s therapeutic factors playing a role in these transformations? Can you provide examples of how they manifested?
3. How did the group dynamics contribute to the transformation you observed? Were there particular interactions that seemed crucial?
4. How did this transformative experience impact your own understanding of group therapy or your role as a therapist?
5. Have you encountered situations where members struggled to experience transformation? What factors contributed to these challenges?

For participants (group members) and therapists

1. Is there anything else you would like to share about your experience of transformation in group therapy that we haven’t covered?
2. How do you think future group therapy sessions could be improved to facilitate more transformative moments

## Appendix B. Participant Quotes by Theme

Emotional Holding: “A Place That Could Hold Me”

“The group hugged my difficulty.”

“I cried and couldn’t say anything. The other member just looked at me with understanding.”

“All the group members hug you and your problem.”

“I felt safe in the group.”

“I didn’t intend to come, but I felt all the warmth and love.”

Risk-Taking and Courage: “Choosing to Step Forward”

“I can either stay silent or finally say it… and when I said it, everything changed.”

“My heart was racing… If I say this, everything might change.”

“He couldn’t say anything and cried in a heartbreaking way.”

“Even though I’m still wearing armor, now it’s not totally closed.”

Internalization of the Group: “Taking the Group Home”

“Even outside the room, I would think: what would they tell me now?”

“My secret angel … the part that speaks kindly because they once did.”

“The members of the group are always with me. I carry them.”

“Even 10 years later I still feel open with the people around me and I want to be close to them.”

Therapist’s Emotional Presence

“She let us see things through our eyes, not hers.”

“It wasn’t what he said. It was how he was with me.”

“Our therapist was amazing … she was the one holding it all together. But always gently.”

“You should be alert to the emotional weather of each member.”

Liberation From Shame: “From Hiding to Being Seen”

“I thought they’d reject me … but they nodded, and I felt human again.”

“I expected disgust or pity … What I met instead was recognition.”

“I was getting a sense of power that I am not the only one.”

“Sometimes you see the other members as a mirror.”
